# BMI-stratified phenotypes of polycystic ovary syndrome: advances in gut microbiota research and personalized management strategies

**DOI:** 10.3389/fendo.2026.1734041

**Published:** 2026-02-06

**Authors:** Bisha Su, Yining Cao, Lin Ma, Jian Huang

**Affiliations:** 1 Fourth Clinical Medical College of Zhejiang Chinese Medical University, Hangzhou, China; 2 Hangzhou Women’s Hospital, Hangzhou, China

**Keywords:** body mass index, gut microbiota, non-obese polycystic ovary syndrome, obese polycysticovary syndrome, personalized treatment, polycystic ovary syndrome

## Abstract

Polycystic ovary syndrome (PCOS) is a heterogeneous endocrine-metabolic disorder affecting 11%-13% of women of reproductive age. Based on body mass index (BMI), patients can be phenotypically classified into obese and non-obese subgroups: the obese PCOS is characterized by insulin resistance, hyperandrogenemia, and metabolic syndrome, with more pronounced metabolic risks; non-obese PCOS primarily manifests as reproductive endocrine dysfunction. In recent years, studies have shown that the Gut microbiota plays a key role in the pathogenesis of PCOS, and dysbiosis in the obese subgroup is generally more pronounced, potentially amplifying metabolic abnormalities through pathways such as short-chain fatty acids, bile acid disturbances, and endotoxin-related low-grade inflammation. This review systematically summarizes the clinically heterogeneous features of BMI-stratified PCOS and its gut microbiota characteristics, with a focus on elucidating the mechanistic differences between obese and non-obese individuals in terms of inflammation, metabolites, and endocrine regulatory pathways. Based on current evidence, individualized intervention strategies targeting different BMI subtypes are proposed, including dietary and lifestyle modifications, interventions with probiotics/prebiotics/synbiotics, and exploration of emerging precision microbiome therapies such as fecal microbiota transplantation. The interaction between BMI and gut microbiota provides new directions for stratified management and personalized treatment of PCOS; however, high-quality longitudinal and interventional studies are still needed to clarify causal relationships and optimize microbiota-targeted strategies.

## 1 Introduction

Polycystic ovary syndrome (PCOS) is a prevalent endocrine and metabolic disorder affecting approximately 11%–13% of women of reproductive age (1), characterized primarily by hyperandrogenism (HA), insulin resistance (IR), and ovulatory dysfunction, often accompanied by an increase in metabolic risks (1, 2). The diagnosis of PCOS remains fundamentally contentious, with the Rotterdam criteria serving as the primary diagnostic framework. These criteria require the presence of at least two of the following conditions: hyperandrogenism, ovulatory dysfunction, or polycystic ovarian morphology (PCOM). In contrast, the National Institutes of Health criteria are more stringent, emphasizing hyperandrogenism and ovulatory dysfunction while not mandating PCOM. Differences in the diagnostic criteria for PCOS not only reflect the complexity of its definition but also directly indicate the significant clinical heterogeneity of the disease: the Rotterdam criteria include more mild cases, whereas the NIH criteria focus on severe phenotypes. One of the important clinical manifestations of this heterogeneity lies in the differentiation of patients’ metabolic characteristics, particularly the significant differences in body mass index (BMI) and obesity status (2).

Traditionally, research and clinical management of PCOS have often used BMI as the primary reference indicator for phenotypic stratification. Despite its known limitations in assessing visceral fat distribution and metabolic health status, BMI, as a simple measure of body fat mass, cannot differentiate between fat and lean mass nor reflect fat distribution, particularly exhibiting inadequacies in evaluating central obesity. In analogous studies, phenotypes such as waist circumference, body fat percentage, and visceral fat area have been demonstrated to more accurately reflect metabolic risk and are closely associated with reproductive endocrine function (3). However, given that BMI is currently an internationally recognized assessment index and the vast majority of existing epidemiological and clinical mechanistic studies employ this metric for grouping, it remains a relatively feasible perspective for integrating evidence from the literature and conducting risk stratification at the present stage (4).

According to the World Health Organization criteria (overweight: BMI ≥ 25 kg/m²; obesity: BMI ≥ 30 kg/m²) (5). The prevalence of obesity among patients with PCOS ranges as high as 38%–88% (6), which is substantially higher than the 18% observed in general women of childbearing age (7). Epidemiological studies further confirm that for every 1% increase in obesity prevalence, the prevalence of PCOS rises by 0.4% (8).

Stratification studies based on BMI have further revealed that patients with PCOS exhibit significant differences in clinical phenotypes and metabolic risks: the obese phenotype of PCOS presents more severe metabolic abnormalities than the non-obese type, often accompanied by hypertension, type 2 diabetes mellitus, and an increased risk of cardiovascular diseases (6, 9), whereas the non-obese type is primarily characterized by reproductive endocrine dysfunction (9, 10). Although some normal-weight patients also exhibit metabolic abnormalities, obesity markedly exacerbates their pathological features. These clinical differences suggest that obese and non-obese PCOS may be driven by distinct pathophysiological mechanisms; however, the molecular basis underlying these related mechanisms remains incompletely elucidated. These unresolved mechanistic issues have sparked interest in the gut microbiota as a potential factor contributing to phenotypic variation among patients with polycystic ovary syndrome.

In recent years, the GM, as a key regulator of metabolism, inflammation, and hormonal homeostasis, has been regarded as an important link for explaining phenotypic heterogeneity in PCOS, particularly the differences between obese and non-obese phenotypes. The GM is acclaimed as the “second genome” of the human body; studies have shown that patients with PCOS exhibit evident dysbiosis, such as reduced α-diversity, elevated Firmicutes/Bacteroidetes ratio, and so forth (11). Further research reveals distinctions in microbial diversity, functional composition, and metabolic byproducts between obese and non-obese PCOS phenotypes. These differences participate in shaping the clinical phenotypes of the disease through multiple mechanistic layers, including inflammatory activation pathways, imbalanced short-chain fatty acid (SCFA) metabolism, bile acid metabolic disturbances, and aberrant signaling within the “gut–brain–ovary axis” (12–14). Therefore, analyzing GM characteristics from the perspective of BMI-based stratification not only facilitates elucidation of the complex pathological mechanisms underlying PCOS, but also provides novel insights for developing microbiota-targeted precision intervention strategies.

## 2 Clinical heterogeneity of PCOS based on BMI

**Figure 1:**
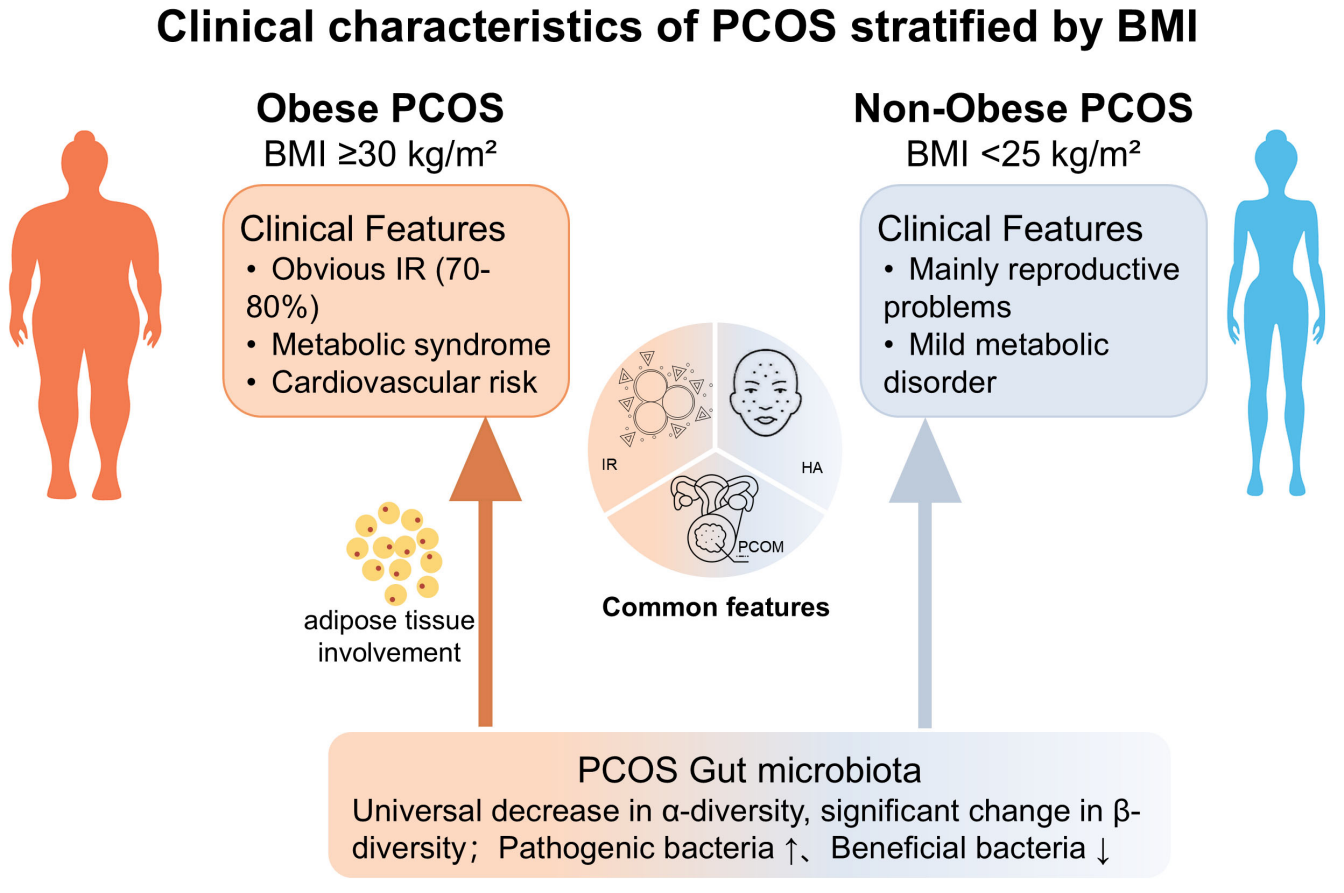
Clinical characteristics of PCOS stratified by BMI.

PCOS is a highly heterogeneous endocrine and metabolic disorder, with its clinical variations partially attributable to differences in BMI. As illustrated in **Figure 1**, obese PCOS patients typically present with pronounced IR, HA, and chronic inflammation, accompanied by elevated risks of metabolic syndrome and cardiovascular diseases. In contrast, non-obese patients, despite exhibiting less prominent metabolic abnormalities, may still experience ovulatory dysfunction and hormonal dysregulation (15), suggesting that these two phenotypes may stem from distinct biological underpinnings.

Epidemiological studies further quantified this difference. Research indicates that the prevalence of IR in obese PCOS patients can reach 70%–80% (16), significantly higher than the 15%–40% observed in non-obese counterparts (17, 18). Regarding HA, obese patients demonstrate more severe clinical manifestations (e.g., hirsutism, acne) and biochemical abnormalities (e.g., elevated free androgen index) (19). Furthermore, in terms of reproductive outcomes, obese PCOS patients face a markedly increased risk of infertility. A 2025 German cross-sectional study revealed that the infertility rate among obese PCOS patients was as high as 77.0%, compared to 53.0% in non-obese patients (20). Multiple large-scale studies have shown that the success rate of *in vitro* fertilization (IVF) in the high-BMI group is reduced by 20% to 40%, and live birth rate, number of oocytes retrieved, and embryo quality are all affected (21, 22). It is noteworthy that pre-pregnancy obesity itself is an independent risk factor for all pregnant women (including those without PCOS), significantly increasing the risks of GDM, gestational hypertension, preeclampsia, etc., and reducing IVF success rates, producing a synergistic effect when coexisting with PCOS. Another meta-analysis further confirmed that PCOS patients had a higher overall BMI (mean difference 1.76 kg/m²); even after adjusting for BMI, PCOS still independently increased the risk of GDM (OR 2.41) and preeclampsia (OR 2.30), suggesting that the pathophysiological abnormalities inherent to PCOS also contribute to the occurrence of pregnancy complications (23).

Further analysis revealed that this risk is dependent on BMI phenotype: in obese PCOS pregnant women, severe insulin resistance and metabolic disturbances are the main drivers of complications such as GDM and gestational hypertension, with high BMI amplifying insulin resistance and significantly exacerbating these risks (21). In non-obese individuals, although the degree of insulin resistance is relatively mild, hyperandrogenism itself is an independent risk factor for GDM and preeclampsia, potentially acting through mechanisms involving abnormal placental function (24). **Table 1** shows the differences between obese and non-obese PCOS in terms of metabolism, endocrinology, and reproduction.

**Table 1:** The metabolic, endocrine, and reproductive differences between obese and non-obese PCOS.

Studies comparing obese and non-obese	Studies comparing obese and non-obese	Studies comparing obese and non-obese
Makhija et al. (2023) cross-sectional study (94)	96 PCOS patients: 66 obese (OP), 30 non−obese.	Metabolism: OP ↑ FINS, HOMAߛIR, postprandial BG (P<0.05); no diff in FPG, TG, HDLߛC. Endocrine: OP ↑ TT, LH/FSH ratio (P<0.001). Reproductive: OP ↑ menstrual irregularity (81.8% vs 23.4%), acne, AN, hirsutism (P<0.05).
Wang et al. (2025) retrospective study (95)	167 PCOS women and 266 non-PCOS controls (subgrouped by normal/high BMI). .	Metabolism: HighߛBMI PCOS group showed milder metabolic abnormalities vs normalߛBMI group (P<0.01). Endocrinology: HighߛBMI ↑ LH, T, AMH, E2 (P<0.05); NormalߛBMI ↑ LH, T, AMH, E2 unchanged. Reproduction: HighߛBMI ↑ miscarriage rate (positively correlated with BMI, P<0.05); no diff in embryo quality or pregnancy rate.
Li et al. (2023) cross-sectional study (96)	255 PCOS patients: 110 obese (OP), 145 non−obese.	Metabolism: OP ↑ FINS, HOMAߛIR, TG, LDLߛC, waist circ., WHR;↓ HDLߛC, HMWA (P<0.01). Endocrine: OP ↑ FAI, AMH; ↓ SHBG, DHEA (P<0.01); T, LH/FSH no diff. Reproductive: OP ↑ uterine artery S/D (P<0.01).
Sachdeva G et al. (2019) Prospective observational study (97)	164 infertile women with PCOS: 124 obese (OP), 40 non−obese.	Metabolism: Obese ↑ FINS, HOMAߛIR, TG, TC, LDLߛC; metabolic syndrome more prevalent (P<0.05). Endocrine: Obese ↑ hirsutism score; T, LH, FSH, LH/FSH no diff. Reproductive: Obese ↑ irregular menses, clomiphene resistance rate higher (58.87% vs 37.5%, P = 0.018).
Chang et al. (2025) case-control study (98)	18 IVF/ICSI patients (9 PCOS, 9 non−PCOS) divided into non−obese and obese groups.	Metabolism: No diff in direct metabolic markers; obese PCOS ↑ FFߛsEVs protein conc. (P = 0.010), positively correlated with BMI (P = 0.006). Endocrine: Overall PCOS ↑ T, AMH, LH, LH/FSH (P<0.05); obesity ↑ HSD3B2, exacerbating hyperandrogenism. Reproductive: Obese PCOS ↑ inflammation/ER stress proteins, trend toward ↓ high-quality embryo rate.

These differences in clinical and reproductive outcomes suggest that obese and non-obese PCOS may have distinct genetic bases. Genome-wide association studies (GWAS) have shown that both share some genetic variants and signaling pathways, but there are also phenotype-specific ones (25). Non-obese PCOS is more likely to exhibit specific associated loci, such as *DENND1A* (rs12000707), *XBP1*, and *LINC02905* (which may affect follicular development via endoplasmic reticulum stress). In contrast, in the combined analysis of the obese type, insulin resistance–related variants (e.g., ERBB4) and another independent signal of *DENND1A* (rs569675099) are more prominent. These loci primarily influence insulin signaling, gonadotropin receptors, and follicular development pathways, highlighting the role of genetic heterogeneity in phenotypic differentiation.

Genetic factors do not act in isolation. Environmental and lifestyle factors such as diet, physical activity, and sleep can amplify genetic effects through gene–environment interactions (26). Patients with different BMI phenotypes show inconsistent treatment responses: the non-obese type is more sensitive to drugs (such as metformin) in terms of reproductive and hormonal improvements, with a greater reduction in testosterone levels compared to the obese type (27, 28). The obese type demonstrates more significant metabolic improvements (body weight, waist circumference, triglycerides, insulin sensitivity, etc.) following weight loss interventions (29, 30). This complementary advantage provides a basis for individualized management.

In summary, BMI classification reveals significant heterogeneity in PCOS at both clinical and genetic levels, and also provides a foundation for risk assessment and individualized management. However, genetic and metabolic differences cannot fully explain the clinical differentiation between the two phenotypes. In recent years, large-scale GWAS and Mendelian randomization (MR) research have shown that current evidence does not support a direct genetic association between specific PCOS susceptibility polymorphisms and gut microbiota typing (31, 32). Most MR effects were attenuated or disappeared after adjusting for BMI, insulin, and sex hormones (33), suggesting that the gut microbiota is more likely to indirectly influence phenotypes through metabolic and endocrine pathways, rather than being directly determined by a single susceptibility locus.In contrast, 16S rRNA and metagenomic sequencing studies have demonstrated that gut microbiota typing is more stably associated with phenotypes such as BMI, IR, and HA (34, 35), and is primarily driven by acquired factors including dietary patterns, obesity, and IR (35, 36). Based on the current data, a framework that better aligns with existing evidence is: the polygenic susceptibility of PCOS influences obesity, IR, and HA under the effect of adverse environmental exposures, subsequently shaping a specific microbial profile; the dysregulated gut microecology then feedbacks to exacerbate metabolic and hormonal abnormalities via pathways such as short-chain fatty acids, lipopolysaccharides, and bile acids. Genetic polymorphisms are more akin to relatively stable “underlying susceptibility,” while the gut microecology serves as a highly plastic “phenotypic amplifier.” Therefore, on the basis of clarifying the clinical and genetic heterogeneity related to BMI, further exploration of differences in microbial composition and function between obese and non-obese PCOS patients from the perspective of gut microecology will help comprehensively understand phenotypic differentiation and provide new targets for individualized intervention.

## 3 Gut microbiota characteristics in PCOS

### 3.1 General characteristics

The human GM consists of approximately 10¹³–10^14^ microorganisms, predominantly bacteria belonging to the phyla *Firmicutes*, *Bacteroidetes*, *Proteobacteria*, and *Actinobacteria* (37, 38). Its composition is influenced by multiple factors including host genetics, age, sex, diet, health status, and medication (39, 40), and it is regarded as a key mediator linking the external environment with host metabolism, potentially playing an important role in the clinical and metabolic heterogeneity of PCOS. This section aims to outline the overall alteration characteristics of the gut microbiota in patients with PCOS, providing a background for subsequent comparisons of microbial differences between PCOS patients with distinct BMI phenotypes (obese vs. non-obese).

Its composition is shaped by host genetics, age, sex, diet, health status, and medication use (39, 40).

Current evidence indicates that the gut ecological structure in patients with PCOS undergoes significant remodeling. A meta-analysis (14 studies, 948 participants) revealed that the overall structure of the GM in patients with PCOS was significantly separated from that of healthy control populations; β-diversity analysis indicated statistically significant differences in microbial composition between the two groups, suggesting a systematic restructuring of the intestinal microbial community in patients with PCOS (41). Conclusions regarding α-diversity remain controversial due to variations in ethnicity or sequencing techniques; however, the overall trend of dysbiosis has been widely recognized. Compared with healthy controls, patients with PCOS generally exhibit a relative reduction in beneficial taxa and an increase in potential pathogenic or opportunistic pathogens, which are closely associated with insulin IR, HA, and chronic low-grade inflammatory states (41, 42). Numerous mechanistic studies have demonstrated that the GM can participate in the onset and maintenance of metabolic abnormalities such as obesity, IR, and inflammation through pathways involving regulation of short-chain fatty acid production, bile acid metabolism, and endotoxemia.

(43, 44). Given the central role of the aforementioned metabolic disturbances in the formation of different PCOS phenotypes (45),the gut microbiome is considered a potential key link connecting metabolic abnormalities with clinical heterogeneity, offering important clues for understanding the differences in clinical manifestations and metabolic features between obese and non-obese patients with PCOS.

### 3.2 Gut microbiota characteristics in obese PCOS

On the basis of overall dysbiosis, microbial alterations are more pronounced in obese patients with PCOS: their α-diversity is significantly reduced, β-diversity differences are prominent, and these changes are closely associated with visceral fat accumulation (46, 47). Specifically, there is a significant increase in lipopolysaccharide (LPS)-producing Gram-negative bacteria (e.g., *Bacteroides, Escherichia/Shigella, Prevotella*), alongside a marked decrease in probiotics (e.g., *Akkermansia, Ruminococcaceae*) (46, 48). Excessive LPS enters the bloodstream under conditions of elevated intestinal barrier permeability, triggering systemic low-grade inflammation, which exacerbates IR and HA (49, 50).

Using PICRUSt2 and KEGG pathway analyses, Bai et al. demonstrated significant enrichment in fatty acid synthesis, carbohydrate metabolism, and inflammation pathways in the gut microbiota of PCOS patients with visceral obesity.These findings suggest that microecological functional alterations may further promote lipid accumulation, chronic inflammation, and metabolic dysfunction (47). Additionally, Yang et al. through a systematic reanalysis of public data, reported that the carbohydrate metabolism pathways of the overall GM in PCOS patients are generally upregulated compared to healthy controls (41). Takeuchi et al. further provided mechanistic insights by demonstrating in both human populations and mouse models that carbohydrate metabolites derived from gut microbes can directly drive IR and inflammation (44), supporting the aforementioned observations.

These findings align with clinical observations: obese PCOS patients are more prone to impaired glucose tolerance or type 2 diabetes, and they exhibit higher carotid intima-media thickness and elevated inflammatory biomarkers, indicating a greater cardiovascular risk (51, 52). It is important to note that most of these associative findings stem from cross-sectional studies, and causality cannot yet be established. Future research should incorporate longitudinal and mechanistic studies to confirm these relationships.

### 3.3 Gut microbiota characteristics in non-obese PCOS

In contrast to obese PCOS, non-obese PCOS patients exhibit less severe GM dysbiosis, with relatively stable α-diversity but still demonstrating distinctive microbial imbalances, such as the presence of *Lactococcus* as a characteristic bacterium, and changes in the composition of functional bacteria such as *Clostridium cluster XIVb* (53, 54). These microbial changes correlate with mild systemic inflammation, impaired insulin sensitivity, and disrupted sex hormone regulation (54, 55).

Phenotypic clustering analyses based on large-scale datasets have identified two major PCOS subtypes: a “metabolic” subtype and a “reproductive” subtype. Notably, patients with the “reproductive” phenotype typically present with lower BMI but more pronounced ovulatory dysfunction and sex hormone abnormalities, closely mirroring the clinical manifestations of non-obese PCOS (56). Further investigations revealed significant enrichment of γ-aminobutyric acid (GABA)-producing microbes—including *Parabacteroides distasonis, Bacteroides fragilis, and Escherichia coli*—in non-obese PCOS, which positively correlate with luteinizing hormone (LH) levels and the LH/FSH ratio. These findings suggest that microbial alterations may influence hypothalamic-pituitary-ovarian (HPO) axis function via the gut-brain axis, thereby contributing to ovulatory dysfunction and hormonal dysregulation (14).

This mechanistic insight aligns with a recent systematic review, which proposed that PCOS-associated GM dysbiosis might impact metabolic and neuroendocrine functions through microbial metabolites (e.g., SCFAs, bile acids, and LPS) as well as gut-brain-HPO axis signaling pathways (36). To facilitate comparison between obese and non-obese PCOS, **Table 2** summarizes the key intestinal microbial alterations discussed above, together with mechanistic clues that will be systematically elaborated in subsequent sections.

**Table 2:** Comparison of gut microbiota changes and related mechanistic pathways between obese and non-obese patients with polycystic ovary syndrome.

Domain	Obese PCOS	Non-obese PCOS	Mechanistic implications
Gut microbiota composition	More pronounced dysbiosis; reduced α-diversity; depletion of SCFA-producing and bileacid–metabolizing bacteria; enrichment of pro-inflammatory taxa (46, 47)	Milder dysbiosis; partial preservation of beneficial taxa (99)	Dysbiosis favors energy harvest and chronic low-grade inflammation, amplifying metabolic disturbances
Short-chain fatty acids	Imbalanced SCFA profile with relatively low butyrate (65, 66)	SCFA profile closer to healthy controls (63)	Impaired gut barrier integrity, reduced GPR41/43 signaling, insulin resistance, and systemic inflammation
ntestinal permeability and LPS	Increased gut permeability with elevated circulating LPS (57)	Limited evidence for clinically relevant barrier dysfunction (62)	Dysbiosis-induced “leaky gut” promotes endotoxemia, triggering inflammation, insulin resistance, and hyperandrogenism
Bile acid metabolism	Altered bile acid composition and disrupted secondary bile acid generation (72)	Subtle or limited bile acid alterations (48, 53)	Dysregulated FXR/TGR5 signaling affects glucose and lipid metabolism and inflammatory responses
Overall phenotype	Metabolic-dominant phenotype (obesity, insulin resistance, inflammation)	Reproductive-dominant phenotype with fewer metabolic abnormalities	Gut microbiota–derived metabolites act as amplifiers linking obesity to endocrine and metabolic dysfunction in PCOS

## 4 Mechanisms linking PCOS and gut microbiota dysbiosis

**Figure 2:**
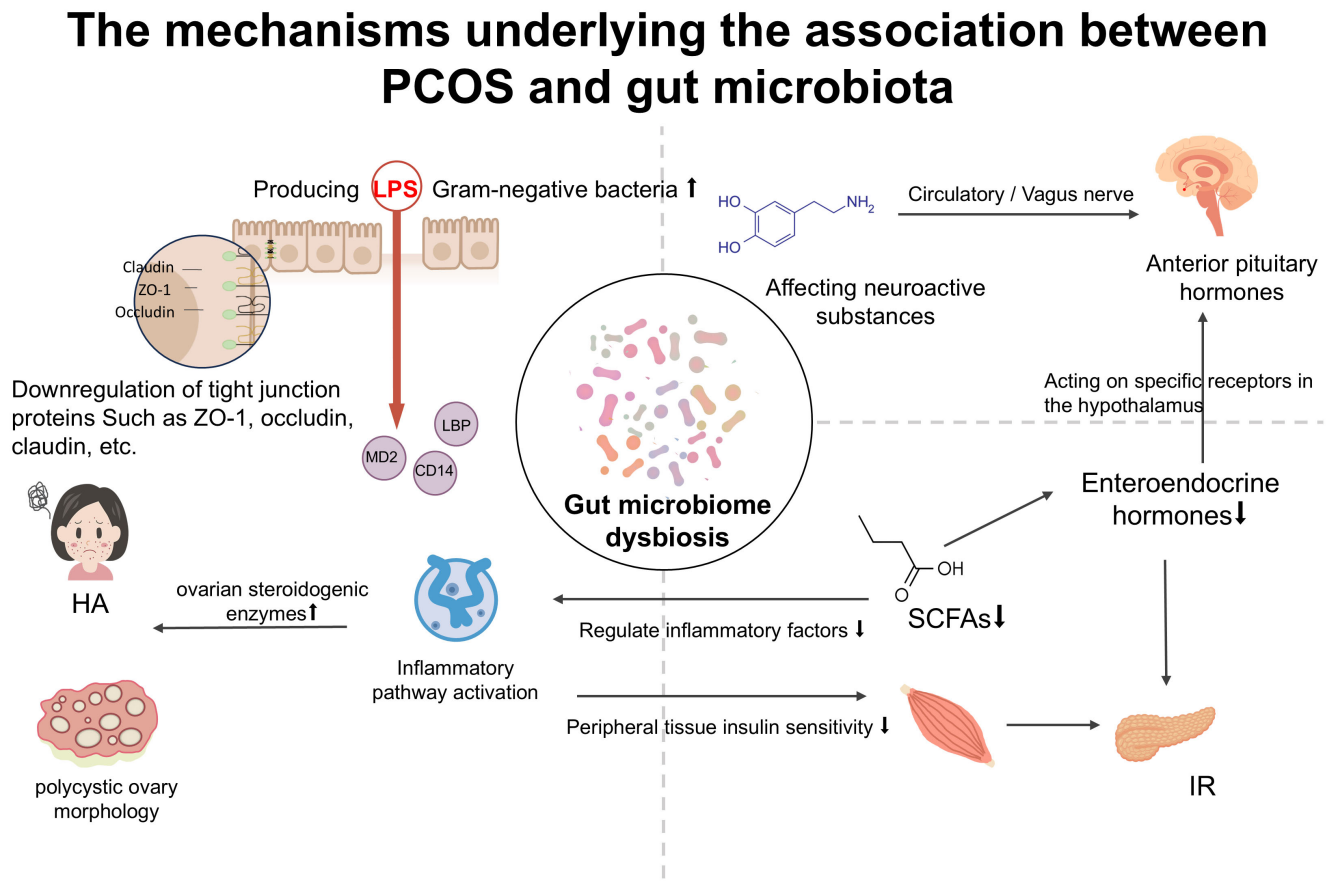
The mechanisms underlying the association between PCOS and gut microbiota.

Existing evidence indicates that GM imbalance is a core pathological feature of PCOS(as shown in **Figure 2**), widely present in patients with different BMI phenotypes. However, obesity, as a significant metabolic state, not only further reshapes the microbial community structure but, more critically, acts as an “amplifier” of pathological pathways. The same microbial signals, in obese versus non-obese host environments, shape heterogeneous clinical phenotypes characterized by metabolic disorders or reproductive axis dysfunction through activation of downstream pathways with different weights.

### 4.1 Endotoxins and low-grade inflammation: BMI-related differences

GM imbalance is prevalent in patients with PCOS, yet it exhibits marked differences across various BMI phenotypes. In obese PCOS, the proportion of Gram-negative bacteria capable of producing LPS is significantly elevated, accompanied by increased intestinal permeability, leading to a notable rise in plasma LPS levels (57). LPS activates downstream inflammatory pathways via Toll-like receptor 4, promoting the release of pro-inflammatory cytokines such as IL-6 and TNF-α, which significantly exacerbates insulin resistance and stimulates ovarian steroidogenic enzymes, thereby contributing to the development of HA (58, 59). Chronic low-grade inflammation in the context of obesity further amplifies this pathway (60, 61).

In contrast, non-obese PCOS patients show a smaller increase in the proportion of LPS-producing bacteria and plasma LPS levels. Studies have indicated that their intestinal barrier function does not differ significantly from that of control groups, or that any changes occur independently of obesity (62). This suggests that the LPS-mediated inflammatory cascade plays a relatively limited role in non-obese individuals, where its effects may be more strongly driven by hyperandrogenism (63). These differences are associated with the amplifying effect of the metabolic environment associated with obesity—such as increased free fatty acids and enhanced oxidative stress—on inflammatory pathways. This finding implies that obesity intensifies the vicious cycle of gut microbiota–inflammation–metabolism, providing a theoretical basis for interventions aimed at restoring intestinal barrier integrity.

### 4.2 Short-chain fatty acid dysregulation: disrupted energy homeostasis

Patients with PCOS commonly exhibit abnormal SCFA metabolism, with differences between obese and non-obese subgroups primarily manifesting in degree and strength of association. A core characteristic is an imbalanced pattern featuring high propionate and acetate alongside low butyrate levels. This pattern is more pronounced in obese PCOS and is closely linked to energy excess, IR, and inflammation, whereas in non-obese PCOS, the changes are milder and show weaker associations with metabolic parameters (63, 64).

In terms of microbial composition, obese PCOS is dominated by taxa associated with metabolic diseases, such as *Veillonellaceaeand and Peptostreptococcaceae* (63); key butyrate-producing species (e.g., *Akkermansia muciniphila, Faecalibacterium prausnitzii*) are reduced, impairing butyrate-mediated protection of the intestinal barrier and anti-inflammatory effects. This is compounded by increased energy recovery from high propionate and acetate levels, further exacerbating IR (65, 66). In non-obese PCOS, some butyrate producers (e.g., *Eubacterium hallii*) are retained, and the magnitude of SCFA abnormalities is smaller, being more strongly influenced by the intrinsic microbial features of PCOS itself (63). High-energy diets and obesity further enrich carbohydrate-preferring bacteria (66), worsening SCFA imbalance, while exogenous butyrate or SCFA mixtures can ameliorate PCOS-related abnormalities, indicating a link between butyrate deficiency and metabolic deterioration (67, 68).

### 4.3 Bile acid signaling axis dysfunction: metabolic–hormonal imbalance

The gut microbiota converts primary bile acids into secondary bile acids via enzymes such as bile salt hydrolase, activating the farnesoid X receptor (FXR)/Takeda G protein-coupled receptor 5 (TGR5) pathways to regulate metabolism and inflammation; disruption of this axis is central to the pathogenesis of PCOS (11). In PCOS overall, conjugated primary bile acids (e.g., glycocholic acid, taurocholic acid) are elevated, whereas conjugated secondary bile acids (e.g., glycochenodeoxycholic acid, tauroursodeoxycholic acid) are reduced, with the latter exacerbating metabolic and reproductive abnormalities by decreasing interleukin-22 secretion (69, 70).

Obesity markedly amplifies this imbalance: in obese PCOS, microbial β-diversity shifts are more pronounced, the Firmicutes/Bacteroidetes ratio is increased, and characteristic taxa (e.g., Coprococcus_2) show stronger correlations with BMI and testosterone (11, 71). Moreover, high-fat diets and obesity further promote accumulation of primary bile acids, suppress the FXR/TGR5 pathway, and exacerbate insulin resistance and lipid metabolism disorders (72). In contrast, the gut microbiota alterations in non-obese PCOS are relatively mild, with metabolic phenotypes more characterized by changes in the abundance of specific bacterial genera (e.g., increased *Lactococcus*), and a less severe dysregulation of the gut microbiota–bile acid axis (48, 53).

Against this background, differences in the metabolic state of hosts with varying BMI may further influence the principal signaling pathways mediated by the microbiota. Beyond bile acid-mediated mechanisms, recent studies have indicated that the intestinal microbiome can also participate in the pathological regulation of PCOS through immune-related pathways; for example, Wu et al. reported that endogenous aryl hydrocarbon receptor antagonists derived from intestinal fungi can inhibit IL-22 secretion, thereby promoting the development of PCOS-like phenotypes (73). Current research suggests that the gut–brain–ovary axis functions more as an “upstream regulator,” wherein, under the context of obesity-related metabolism and inflammation, it remodels central energy sensing and stress responses via gut hormones and microbiota–inflammation signaling pathways, thereby indirectly regulating gonadotropin-releasing hormone (GnRH) pulsatility and HPO axis function (74). In contrast, in non-obese PCOS, the amplifying effect of BMI on central metabolic signals is relatively limited, and reproductive abnormalities are more likely to reflect intrinsic functional imbalances of the classical HPO axis at the pituitary and ovarian levels, rather than altered rhythms of GnRH neurons themselves, with a hyperandrogenic state playing a dominant role (75, 76). It should be noted that clinical studies directly stratifying by BMI and simultaneously comparing the relative contributions of the classical HPO axis and the gut–brain–ovary axis remain limited; existing evidence largely derives from mechanistic studies or single-axis perspectives, and the relevant causal relationships and dynamic regulatory patterns still require further elucidation.

In summary, the microbiota-related mechanisms of PCOS stratified by BMI demonstrate distinct heterogeneity: obesity drives a multi-pathway synergistic pathological cycle by amplifying microbiota-mediated inflammation, metabolic disorders, and dysregulation of hormonal regulatory axes; non-obese PCOS is characterized by mild microbial metabolic abnormalities and intrinsic hormonal axis imbalances. This phenotype-specific mechanism provides key targets for differential management strategies: for obese PCOS, focus can be placed on intestinal barrier repair, inflammation inhibition, and regulation of microbial metabolic balance; for non-obese PCOS, priority should be given to improving hormonal axis function and intervening against hyperandrogenism. Based on the aforementioned mechanistic differences, the next section will further explore specific strategies for stratified intervention in PCOS and the clinical evidence supporting them.

## 5 Differentiated treatment strategies based on gut microbiota

With the increasingly clear role of the GM in the pathogenesis of PCOS, microecological modulation–based interventions have gradually emerged as a new area of research focus. Given the significant differences between obese and non-obese PCOS patients in terms of GM structure, metabolic burden, inflammatory status, and endocrine dysregulation, developing differentiated microecological intervention strategies guided by BMI stratification holds promise for achieving more precise and individualized disease management.

### 5.1 BMI-stratified overall framework for microbiota interventions

BMI stratification not only reflects the body fat distribution and metabolic status of patients with PCOS, but also partially determines the characteristics of their GM dysbiosis and their response to interventions. Obese PCOS is typically accompanied by marked IR, chronic low-grade inflammation, and metabolic disturbances, with the primary goals of microbiome-based interventions focusing on alleviating metabolic burden, enhancing insulin sensitivity, and suppressing inflammatory responses. In contrast, non-obese PCOS patients, despite having normal body weight, often present with ovulatory dysfunction and hyperandrogenism, necessitating an intervention strategy that prioritizes maintaining microbial homeostasis, modulating gut–brain–ovary axis function, and improving the endocrine environment. Against this backdrop, the positioning and prioritization of microbiome-modulating approaches vary markedly across different BMI phenotypes, warranting differentiation in therapeutic planning.

### 5.2 Obese PCOS: metabolism improvement as the core

The core of microecological interventions for obese PCOS lies in alleviating metabolic disturbances through modification of gut microbiota structure. Lifestyle modifications constitute the foundational approach; dietary patterns characterized by low glycemic index, Mediterranean-style diets, and fiber-rich regimens have been demonstrated to enhance beneficial bacterial abundance, increase SCFA production, and attenuate inflammatory responses (77, 78). Through caloric restriction combined with high fiber intake, both weight reduction and improvements in IR and multiple metabolic markers can be achieved, thereby indirectly modulating GM dysbiosis (79, 80).

Building upon this foundation, probiotics, prebiotics, and synbiotics serve as direct GM-targeted interventions and have shown consistent metabolic benefits in obese PCOS. Multiple clinical studies indicate that probiotic supplementation improves gut microbiota diversity, reduces the proportion of inflammation-associated taxa, and exerts positive effects on lowering BMI, ameliorating IR, and mitigating dyslipidemia (81, 82). Prebiotics such as fructooligosaccharides, galactooligosaccharides, and resistant dextrin selectively promote the proliferation of lactobacilli and bifidobacteria, augment SCFA generation, and enhance metabolic homeostasis via modulation of gut-derived hormones such as glucagon-like peptide-1 (GLP-1) and peptide YY (PYY) (82, 83). Synbiotics have demonstrated more pronounced effects on weight management, metabolic improvement, and hormonal balance in women with obese PCOS (84, 85).

Moreover, the efficacy of certain metabolic medications may partly depend on GM remodeling. Metformin, in addition to improving IR, exerts metabolic regulatory effects through increasing beneficial taxa such as Akkermansia, with particularly notable efficacy in obese PCOS (86). Furthermore, GLP-1 receptor agonists and sodium-glucose cotransporter 2 inhibitors not only facilitate weight loss but also improve reproductive function in obese PCOS patients, suggesting that the combined application of pharmacotherapy and microecological modulation possesses considerable clinical potential (87, 88).

### 5.3 Non-obese PCOS: endocrine and reproductive function recovery as the core​​

Non-obese patients with PCOS generally do not require strict energy restriction, and their dietary interventions should place greater emphasis on diet quality and the appropriate provision of microbial substrates. Dietary patterns rich in whole grains, dietary fiber, and oligosaccharides help maintain GM homeostasis and may improve the endocrine environment by modulating the gut–brain–ovary axis (89).

Current research indicates that probiotic supplementation in non-obese PCOS patients is more likely to yield reproductive and hormonal benefits, such as restoration of menstrual cycles, improved ovulation function, and reduced androgen levels (90). Although studies on prebiotics and synbiotics in non-obese PCOS are relatively limited, preliminary evidence suggests they may indirectly promote hormonal balance and reproductive function recovery by improving insulin sensitivity and reducing inflammation (82). However, existing research is constrained by small sample sizes, high intervention heterogeneity, and a predominant focus on metabolic endpoints, leaving the long-term efficacy and underlying mechanisms requiring further validation.

### 5.4 Emerging microbiota-targeted therapies: FMT and future directions​

Fecal microbiota transplantation (FMT), by reconstructing the intestinal microecological structure, has demonstrated certain potential in inflammatory bowel disease and metabolic disorders. Animal studies have shown that FMT can ameliorate hyperandrogenism and ovulatory dysfunction in PCOS model animals (91). However, current clinical evidence for FMT in PCOS remains limited, and its safety, donor screening criteria, and long-term efficacy are not yet clearly established. At present, FMT is more suitable as an exploratory strategy for refractory or special cases, rather than a routine treatment modality.

### 5.5 BMI-stratified integrated microbiota-targeted therapeutic pathway

Based on the available evidence, microecological interventions for PCOS stratified by BMI can follow distinct therapeutic pathways: for obese PCOS patients, the focus should sequentially be placed on lifestyle modification, prebiotic and synbiotic microecological regulation, and the combination of metabolic drugs, with advanced microecological therapies explored when necessary. For non-obese PCOS patients, greater emphasis should be given to optimizing dietary structure, along with probiotic or synbiotic interventions, integrated with traditional treatments aimed at restoring endocrine and ovulatory function. This stratified strategy helps enhance the targeting of microecological interventions, providing a theoretical basis for the precise, individualized management of PCOS.

## 6 Non-obese PCOS: limited evidence and research recommendations

Compared with obese PCOS, research and evidence regarding the mechanisms of GM involvement in non-obese PCOS remain notably insufficient (2). Existing literature predominantly focuses on the GM dysbiosis characteristics of obese patients (42), whereas studies on non-obese PCOS are mostly at the exploratory stage, with limited sample sizes and inadequate stratification, resulting in a lack of systematic elucidation of its potentially specific mechanisms. For example, possible pathways in non-obese PCOS involving gut–brain axis signaling abnormalities, changes in specific microbial taxa, and GM-mediated modulation of HPO axis function via tryptophan metabolism currently yield inconsistent results due to study designs that are mainly cross-sectional and based on small samples, making it difficult to draw stable conclusions.

From a research perspective, the insufficiency of evidence is mainly reflected in the following aspects: the obese phenotype dominates research cohorts, and there is a lack of GM data stratified according to BMI or clinical subtypes (92); phenotypic characterization often relies on a binary definition of PCOS presence or absence, without adequately distinguishing reproductive-type from metabolic-type features (93); and GM is rarely incorporated into a unified framework integrating genetic and metabolic indicators (36). These factors collectively limit the ability to determine the position and causality of GM effects in non-obese PCOS, and to some extent marginalize management strategies for this population. In addition, current microecological intervention studies targeting different BMI phenotypes still lack specificity assessments and long-term efficacy data, further delaying the establishment of individualized treatment plans for non-obese PCOS.

Therefore, future research should treat non-obese PCOS as an independent subject of investigation, incorporate BMI and clinical subtype stratification in cohort construction, and combine longitudinal follow-up or interventional designs to systematically evaluate the dynamic relationship between GM and hormonal and metabolic indicators. Simultaneously, GM should be integrated into multi-omics frameworks and validated across diverse populations and dietary backgrounds, so as to progressively fill the evidence gap regarding GM in non-obese PCOS and develop more targeted management and intervention strategies for this subgroup (2).

## 7 Discussion

The clinical heterogeneity of PCOS has been widely recognized; however, existing classification systems primarily rely on horizontal categorization based on clinical manifestations, such as hyperandrogenemia, ovulatory dysfunction, or PCOM, while systematic integration of metabolic background differences among patients remains relatively insufficient. This review, based on a comprehensive analysis stratified by BMI, demonstrates that BMI not only reflects variations in body fat distribution and metabolic status but also participates, to a certain extent, in shaping the pathophysiological patterns dominated by different PCOS phenotypes. Integrating the available evidence, the core argument proposed in this review is that BMI is not merely a clinical stratification marker for PCOS but serves as a critical determinant that drives phenotype-specific alterations in gut microbiota by shaping distinct adverse biological pathways—either metabolism–inflammation–dominant or reproductive axis dysfunction–dominant—thereby providing a biological rationale for stratified intervention strategies.

Based on current evidence, obese PCOS is typically characterized by insulin resistance, hyperandrogenism, and metabolic syndrome, with more pronounced gut microbiota dysbiosis manifested as reduced microbial diversity, enrichment of pro-inflammatory taxa, and disturbances in short-chain fatty acid and bile acid metabolism. These alterations further exacerbate metabolic burden through pathways involving chronic low-grade inflammation, abnormal energy metabolism, and endocrine regulatory imbalance. In contrast, non-obese PCOS patients exhibit relatively milder overall metabolic disturbances, yet specific changes in gut microbiota composition and function persist; these changes may influence hormonal regulation and ovulatory function via the gut–brain–ovary axis. Such differences suggest that alterations in gut microbiota across BMI phenotypes are not simply quantitative distinctions but may contribute to pathophysiological divergence determining the dominant disease pathways. It should be noted that metabolic, inflammatory, and neuroendocrine pathways are not mutually exclusive but exhibit varying relative weights within different BMI-associated metabolic contexts.

On this basis, this review systematically integrates the association between PCOS and gut microbiota from the perspective of BMI stratification. Unlike previous reviews that primarily analyzed the overall PCOS population and focused mainly on global microbiota dysbiosis, this review expands current understanding in the following aspects: (1) systematic integration of BMI stratification, clinical phenotypic characteristics, and differences in gut microbiota composition and function; (2) recognition of non-obese PCOS as a subtype with independent biological features, rather than a milder manifestation of obesity-related phenotypes; (3) emphasis on the pivotal role of BMI-associated metabolic environments in shaping microbiota characteristics and their downstream metabolic–endocrine effects, linking these to stratified intervention strategies. This perspective facilitates a deeper understanding of the biological basis underlying PCOS phenotypic heterogeneity.

Despite the growing number of studies on the relationship between PCOS and gut microbiota in recent years, several limitations warrant attention. First, most studies have limited sample sizes and are predominantly cross-sectional in design, making it difficult to establish causal relationships between gut microbiota alterations and disease phenotypes. Second, research subjects are mainly concentrated in Asian and European–American populations; differences in the metabolic significance of BMI across ethnicities and dietary backgrounds may affect the generalizability of findings. Additionally, existing studies tend to focus on obese PCOS, while systematic evidence regarding microbiota-specific mechanisms and clinical implications in non-obese PCOS remains lacking (see Section VI for details).

Future research should conduct more refined longitudinal and interventional studies based on BMI stratification to dynamically assess the relationship between gut microbiota changes and metabolic and reproductive outcomes. Concurrently, integration of multi-omics data will help clarify the interactions among BMI, gut microbiota, and PCOS phenotypes. Building on these insights, formulating differentiated microecological intervention strategies according to BMI phenotypes may enhance the precision and clinical feasibility of individualized management for PCOS.
